# Dexamethasone induces an imbalanced fetal-placental-maternal bile acid circulation: involvement of placental transporters

**DOI:** 10.1186/s12916-021-01957-y

**Published:** 2021-04-07

**Authors:** Wen Huang, Jin Zhou, Juanjuan Guo, Wen Hu, Guanghui Chen, Bin Li, Yajie Wen, Yimin Jiang, Kaili Fu, Huichang Bi, Yuanzhen Zhang, Hui Wang

**Affiliations:** 1 Department of Obstetrics and Gynaecology, Zhongnan Hospital of Wuhan University, Wuhan, 430071 China; 2 Department of Pharmacology, Basic Medical School of Wuhan University, Wuhan, 430071 China; 3 Hubei Provincial Key Laboratory of Developmentally Originated Diseases, Wuhan, 430071 China; 4 School of Pharmaceutical Sciences, Sun Yat-sen University, Guangzhou, 510006 China

**Keywords:** Dexamethasone, Placenta, Bile acids, Transporters, Glucocorticoid receptor, Farnesoid X receptor

## Abstract

**Background:**

The use of prenatal dexamethasone remains controversial. Our recent studies found that prenatal dexamethasone exposure can induce maternal intrahepatic cholestasis and have a lasting adverse influence on bile acid (BA) metabolism in the offspring. The purpose of this study was to investigate the effects of dexamethasone on fetal-placental-maternal BA circulation during the intrauterine period, as well as its placental mechanism.

**Methods:**

Clinical data and human placentas were collected and analyzed. Pregnant Wistar rats were injected subcutaneously with dexamethasone (0.2 mg/kg per day) from gestational day 9 to 20. The metabolomic spectra of BAs in maternal and fetal rat serum were determined by LC-MS. Human and rat placentas were collected for histological and gene expression analysis. BeWo human placental cell line was treated with dexamethasone (20–500 nM).

**Results:**

Human male neonates born after prenatal dexamethasone treatment showed an increased serum BA level while no significant change was observed in females. Moreover, the expression of organic anion transporter polypeptide-related protein 2B1 (OATP2B1) and breast cancer resistance protein (BCRP) in the male neonates’ placenta was decreased, while multidrug resistance-associated protein 4 (MRP4) was upregulated. In experimental rats, dexamethasone increased male but decreased female fetal serum total bile acid (TBA) level. LC-MS revealed that primary BAs were the major component that increased in both male and female fetal serum, and all kinds of BAs were significantly increased in maternal serum. The expression of Oatp2b1 and Bcrp were reduced, while Mrp4 expression was increased in the dexamethasone-treated rat placentas. Moreover, dexamethasone increased glucocorticoid receptor (GR) expression and decreased farnesoid X receptor (FXR) expression in the rat placenta. In BeWo cells, dexamethasone induced GR translocation into the nucleus; decreased FXR, OATP2B1, and BCRP expression; and increased MRP4 expression. Furthermore, GR was verified to mediate the downregulation of OATP2B1, while FXR mediated dexamethasone-altered expression of BCRP and MRP4.

**Conclusions:**

By affecting placental BA transporters, dexamethasone induces an imbalanced fetal-placental-maternal BA circulation, as showed by the increase of primary BA levels in the fetal serum. This study provides an important experimental and theoretical basis for elucidating the mechanism of dexamethasone-induced alteration of maternal and fetal BA metabolism and for exploring early prevention and treatment strategies.

## Background

Bile acids (BAs) are physiological cholesterol-derived agents for hydrophobic compound excretion and fat absorption. They can also serve as important signal molecules to maintain the balance of glucose, lipid, and energy metabolism in the body [1]. In utero, BAs can also promote the proliferation of hematopoietic stem cells to maintain the development of the fetal liver [2]. Hydrophobic BAs are also called toxic BAs, which mainly refer to the primary unconjugated BAs, such as cholic acid (CA), chenodeoxycholic acid (CDCA), and muricholic acid (MCA). Toxic BAs can disrupt the integrity of cell membranes by their detergent effect on lipid components. Studies found that high BA levels can cause oxidative damage and apoptosis in the placenta and fetal liver [3, 4] due to their detergent properties. An epidemiological study has shown that fetuses of women with intrahepatic cholestasis during pregnancy are exposed to high intrauterine BA levels and are at an increased risk of obesity and abnormal lipid metabolism as adults [5]. Therefore, normal BA concentration is crucial for maternal health and offspring’s development.

The fetal liver is known to synthesize primary unconjugated BAs. Differently from the adults, BAs are synthesized in the fetal liver via the alternative pathway mediated by cholesterol 27α-hydroxylase (CYP27A1), while cholesterol 7α-hydroxylase (CYP7A1) activity is undetectable in fetuses less than 30 weeks of gestation [6]. Unconjugated BAs are then combined with taurine by hepatic bile acid-CoA ligase (BACL) (encoded by *Slc27a5*) to form a variety of conjugated BAs, increasing their water solubility and excretion [7]. Due to the immaturity of the fetal liver and the lack of intestinal microflora [8, 9], the entero-hepatic circulation of BAs is absent in the fetus, and the metabolic process of BAs is incomplete. Thus, primary BAs are predominant components in fetal BA pool and are transferred to the mother across the placenta and converted into secondary BAs under the maternal intestinal microflora [10, 11]. Some researchers have found that the secondary BAs in the maternal circulation can be transported back to the fetus through the placenta [12]. Therefore, fetal blood BA levels are maintained by the physiological “fetal-placental-maternal BA circulation.”

The placenta is an important organ for the exchange of BAs between the maternal and fetal blood, and an impaired placental BA transportation has been found to contribute to imbalanced fetal blood BAs [13]. A variety of BA transporters and related nuclear receptors with regulatory effects have been identified on the placenta [14, 15]. For example, the organic anion transporter peptides 2B1 (OATP2B1) was identified in the fetal- (basal) facing trophoblast plasma membrane (bTPM) where it mediates the uptake of BAs from fetal serum in an acidic environment [16, 17], and the expression of Oatp2b1 in rat placenta increases with gestation [18]. Breast cancer resistance protein (BCRP), as one of the important ABC pumps in the placenta, plays a crucial role in the excretion of BAs into the maternal circulation [19]. Additionally, multidrug resistance-associated protein 4 (MPR4) can favor BA flow from the mother to the fetus under the maternal cholestatic condition [11]. Nuclear receptors are known to regulate the BA metabolism in the organism [20, 21]. Investigators have confirmed the expression of several important nuclear receptors in the rat and human placenta, such as the farnesoid X receptor (FXR) [22, 23].

Dexamethasone, as a synthetic glucocorticoid, is widely used in the case of threatened preterm labor to promote fetal lung maturation and reduce premature infant mortality. A survey conducted by the World Health Organization (WHO), including 29 countries in Africa, Asia, Latin America, and the Middle East, showed that an average of 54% of pregnancies received synthetic glucocorticoid treatment, up to 94% [24]. However, the use of prenatal dexamethasone remains controversial. An increasing number of studies have confirmed that prenatal dexamethasone administration reduces fetal birth weight [25] and induces developmental toxicity and related diseases in various organs in adult offspring [26–28]. A previous clinical study found that dexamethasone treatment for premature labor can increase BA synthesis rate in neonates [29]. Our recent series of animal studies have shown that prenatal dexamethasone exposure (PDE) can induce maternal intrahepatic cholestasis and have a lasting adverse influence on BA metabolism in the offspring [30, 31]. These studies indicate that PDE can alter BA metabolism in the maternal and fetal circulation. However, little is known about the effects of prenatal dexamethasone on the fetal-placental-maternal BA circulation.

In this study, we aimed to verify the effects of PDE on the fetal-placental-maternal BA circulation both in humans and rats and identify the key factors contributing to the imbalanced BA circulation. Because of the essential role of the placenta in the exchange of BAs between the mother and fetus, this study further illustrates the effect and mechanism of the placenta in the PDE-altered fetal-placental-maternal BA circulation. This study provides a theoretical and experimental basis for the rational application of dexamethasone during pregnancy.

## Methods

### Drugs and reagents

Dexamethasone (No. H42020019) was purchased from Shuanghe Pharmaceutical Co., Ltd. (Wuhan, China). Isoflurane was obtained from Baxter Healthcare Co., Ltd. (Deerfield, IL, USA). Total bile acid (TBA) assay kit was purchased from Jiancheng Bioengineering Institute (Nanjing, China). Dulbecco’s modified eagle medium (DMEM) and fetal bovine serum (FBS) were supplied by Gibco (St. Louis, MO, USA). Reverse transcription kits and real-time quantitative polymerase chain reaction (RT-qPCR) kits (Q223) were purchased from Takara Biotech Co., Ltd. (Dalian, China), and primers were synthesized by Sangon Biotech Co., Ltd. (Shanghai, China). Primary antibodies including farnesoid X receptor (FXR) (sc-25309), glucocorticoid receptor (GR) (sc-56851), and glyceraldehyde-3-phosphate dehydrogenase (GAPDH) (sc-47724) were purchased from Santa Cruz Biotech Co., Ltd. (Santa Cruz, USA); organic anion transporter polypeptide-related protein 2b1 (SLCO2B1) (abs124881a), breast cancer resistance protein (BCRP) (abs102521), and multidrug resistance-associated proteins 4 (MRP4) (abs122912a) were purchased from Absin Biotech Co., Ltd. (Shanghai, China). Mifepristone (M8064) was purchased from Sigma-Aldrich Co., Ltd. (St. Louis, MO, USA). FXR agonist-GW4064 (M2018) was obtained from Abmole (Shanghai, China). Chenodeoxycholic acid (MB5978) and taurocholic acid (MB5972) were purchased from Dalian Meilun Biotech Co., Ltd. (Dalian, China). The other reagents for experiments were of analytical grade.

### Human subjects and placental tissue collection

This study was approved by the Research Committee for Human Subjects, Zhongnan Hospital of Wuhan University, China (No. 2016016). A retrospective study was conducted with medical records of pregnant women at risk of imminent preterm birth and their neonates from June 1 to December 1, 2017. The following criteria were required for the inclusion: (1) neonates requiring admission to the neonatal intensive care unit; (2) pregnancies that received dexamethasone treatment between 34 and 36 6/7 weeks of gestation; or (3) received treatment of dexamethasone at 24 to 33 6/7 weeks of gestation but delivered beyond 24 h to 7 days; or (4) those who delivered between 34 and 37 weeks without administration of dexamethasone. Seventy-six male neonates and forty-eight female neonates recruited in this study were categorized into two groups for each sex: the control group without dexamethasone administration and the dexamethasone group with a single course of treatment. Demographic and pregnancy characteristics of recruited subjects were collected. We compared clinical assessment of imminent preterm birth, maternal age, gestational age, birth weight of neonates, Apgar score of 5 min, and fetal serum BA levels between two groups. Apgar score can describe the condition of the newborn infant and, when properly applied, serves as a tool to predict infants’ outcomes [32]. To avoid confusion of the diseases and multiple pregnancies on the results, pregnant women with abnormal liver functions, with severe obstetrics complications (such as preeclampsia, gestational diabetes, and chorioamnionitis), and with multiple pregnancies were excluded.

To avoid artifact from the effects of labor, only placental samples obtained from women who underwent cesarean sections were selected. The fetal indications, such as malpresentation, fetal distress, and nuchal cord, contributed most to the cesarean delivery rate in China [33]. Ten male neonatal placentas, five of the control group and five of the prenatal dexamethasone therapy group, were finally recruited. Maternal age, gestational age, birth weight of neonates, and fetal serum TBA levels of recruited subjects were collected. All samples were collected immediately after the cesarean section, as previously reported [34]. Placental cotyledons were dissected at the middle zone and washed thoroughly with saline after amniotic membranes, decidua, and connective tissues had been removed. The placenta samples used for immunohistochemistry were fixed in 4% paraformaldehyde. All participating pregnant women gave written informed consent. Data analysis and human placenta collection were conducted blindly and independently by three investigators.

### Animals and treatment

Specific pathogen-free (SPF) Wistar rats for experiments (No. 2015-0018, certification number 42000600014526, license number SCXK (Hubei)) were obtained from the Experimental Center of the Hubei Medical Scientific Academy (Wuhan, China). The body weights of female and male rats are 200 ± 20 g and 280 ± 20 g, respectively. All animal experimental procedures were conducted following the Guidelines for the Care and Use of Laboratory Animals of the Chinese Animal Welfare Committee. The Committee on the Ethics of Animal Experiments of the Wuhan University School of Medicine approved the protocol (Permit 14016). All efforts were made to minimize the number of animals used and their suffering. Animals were housed in cages with padding in an air-conditioned room under standard conditions (room temperature, 18–22 °C; humidity, 40–60%; light cycle, 12-h light-dark cycle) and allowed free access to rat chow and tap water. All rats were allowed to acclimate 1 week before experimentation. Virgin female and male rats were subjected to experimental conditions. Overnight, two female rats were placed together with one male rat in a cage for mating. The appearance of sperm in vaginal smears was designated as the gestational day (GD) 0. As previously described [35], the PDE group was treated with 0.2 mg/kg day dexamethasone subcutaneously from GD9 to GD20, respectively. The control group was sham-treated with saline. The National Institutes of Health (NIH) recommended that prenatal dexamethasone treatment (therapeutic use) be set at 6 mg intramuscular injection per 12 h (four times for one course) [36, 37], which is equivalent to 0.2 mg/kg day of dexamethasone for a 60-kg person. Using the dose conversion between humans and rats (human to rat 1:6.17) [38], the dexamethasone doses used in this study can be reached in human exposures. On GD20, 1 h after the last injection of dexamethasone, the pregnant rats were anesthetized with 3–3.5% isoflurane and then sacrificed. There were 11 pregnant rats in each group, and the litter size was set to 8–15 rats. Maternal and fetal blood was collected through cutting the bilateral carotid arteries and catching drops of the blood. The blood samples of fetuses from each litter were pooled as one sample according to gender, respectively. Serum samples were obtained from blood by centrifugation at 4000 rpm, 4 °C for 15 min. After the collection of maternl blood, the anesthetized rats were sacrificed by cervical dislocation. The fetal livers and placentas from the same dam were collected and pooled together according to the gender and divided into four groups: male control, female control, male PDE, and female PDE groups. All samples were transferred to liquid nitrogen immediately, followed by storage at − 80 °C for total RNA extract and total protein extract. Drug preparation, animal treatment, and data collection were conducted blindly and independently by three investigators.

### BA analysis

Serum total bile acid (TBA) concentration was measured using a commercially available kit following the manufacturer’s instructions. To analyze the metabolomic spectrum of BAs, serum samples were thawed, and 20 μl was added to a tube containing 180 μl of 67% aqueous acetonitrile. The samples were vortexed for 30 s and centrifuged at 18,000×*g* for 20 min at 4 °C to remove proteins. Then, the samples were analyzed using a previously described method for BA pattern measurement by LC-MS [39]. For detecting the BA concentration in the placenta, 50 mg of placenta tissue was selected and homogenized with an electric high-speed homogenizer (KINEMATICA, Switzerland) in 500 μl of lysis buffer. The mixture was then centrifuged at 2000×*g*, 5 min. Then, 10 μl of the supernatant was added to a glass tube containing a working liquid for the TBA assay. After incubating for 20 min at 37 °C, the absorbance was measured at 550 nm. The protein concentration was determined, and the BA concentration was expressed as μmol/mg protein.

### Immunohistochemistry detection

Placental tissues of different treatments were fixed in a 4% paraformaldehyde solution overnight at room temperature and processed with the paraffin sectioning technique. Paraffin-embedded tissues were cut into 5-μm-thick serial sections, and the slices were dewaxed and washed with PBS. After antigen retrieval, placental sections were blocked in 5% blocking serum at room temperature for 30 min and probed with primary antibodies overnight at 4 °C, including rabbit anti-SLCO2B1 (1:50 dilutions), rabbit anti-BCRP (1:50 dilutions), and rabbit anti-MRP4 (1:100 dilutions) and then incubated with a biotinylated secondary antibody and an avidin-biotinylated horseradish peroxidase complex solution according to the manufacturer’s directions. Finally, peroxidase activity was determined with a diaminobenzidine (DAB) staining kit. Images of three random fields from each section were captured by the Photo Imaging System (Nikon H550S, Tokyo, Japan) at × 400 magnification. In brief, the original JPEG images were converted to 8-bit images. After that, they were auto-thresholded to binary photos by using the “Make Binary” function in ImageJ. Then, regions of interest (ROIs) were selected around each DAPI-stained nucleus by the “Add to Manager” function. These selections only enclose the cellular membranes where the BA transporter protein was found. After that, the areas of ROIs in each of the binary images were calculated by the “Analyze Particle” function, and the sum of integrated optical density (IOD SUM) of BA transporters in the cells was measured after background fluorescence was dislodged by “Subtract Background” function. Finally, the mean IOD was calculated as a ratio of IOD SUM relative to the area. All the samples were scored blindly and independently by three investigators.

### Cell culture and treatment

The human choriocarcinoma cell line BeWo, which has been widely used in in vitro models for studying placental transport function [40], was obtained from the China Centre for Type Culture Collection (Wuhan, China). The cells were seeded in a cell culture flask (25 cm^2^, Corning, USA), which contained DMEM supplemented with 10% fetal bovine serum and 0.1% penicillin/streptomycin, and cultured in a 5% CO_2_ humidified incubator at 37 °C. According to our previous animal study [41] and another published clinical data [42], the dexamethasone concentration of maternal serum was 846 ± 214 nM when the pregnant rats were subcutaneously injected with 0.2 mg/kg day dexamethasone and 101.7 ± 19.2 ng/mL (molecular weight of dexamethasone is 392.4, this concentration was equal to 260 ± 49 nM) after intramuscular injection of 6 mg dexamethasone in third-trimester pregnant women. Thus, we treated BeWo with 20, 100, and 500 nM of dexamethasone for 5 days, respectively. Cells were cultured in 96-well plates in the presence of dexamethasone at different concentrations (0, 20, 100, and 500 nM) for 5 days. MTS assay was conducted to detect the cytotoxicity of dexamethasone on BeWo cells following the manufacturer’s protocol. Absorption intensity was measured at 490 nm using a microplate reader (TECAN, Australia).

To determine the effect of dexamethasone on placental BA transport, the cells were treated with 20, 100, and 500 nM of dexamethasone for 5 days. To confirm the signaling pathway, a GR antagonist RU486, GR siRNA, and FXR agonist GW4064 were used. Briefly, the BeWo cells were cultured in 6-well plates at a density of 5000 cells/well. After reaching 30–50% confluence, cells were treated with RU486 (10 μM), GR siRNA (100 nM), and GW4064 (10 μM), with or without dexamethasone (500 nM) for 5 days, respectively. The culture medium was replaced every other day. Then, the cells were collected for the subsequent analysis. As to the siRNA-mediated knockdown of GR expression, three sequences of GR siRNA were designed and synthesized by Hippo Biotech Co., Ltd. (Huzhou, China). And we screened out the most effective sequence: 5′-GGAGAUGACAACUUGACUUTT-3′ (sense sequences) and 5′-AAGUCAAGUUGUCAUCUCCTT-3′ (antisense sequences). The siRNA-mediated knockdown of GR expression was performed after the culture in 12-well plates for 24 h under standard conditions; BeWo cells were transfected with siRNA targeting human GR and negative control siRNA (sequences: sense: 5′-UUCUCCGAACGUGUCACGUTT-3′; antisense: 5′-ACGUGACACGUUCGGAGAATT-3′) at a final concentration of 100 nM using Lipofectamine 3000 (Invitrogen, Carlsbad, CA, USA) according to the manufacturer’s introductions. To verify GR knockdown, we performed RT-qPCR after 24-h transfection. To confirm the role of GR in the signaling pathway, the transfected cells were then incubated with or without 500 nM dexamethasone for 3 days. And then, cells were collected for the subsequent analysis.

### In vitro transport experiments

Transwell culture plates (Corning, USA) were used to study the effects of dexamethasone on bile acid transport activity via the placenta in vitro. BeWo cells were seeded at a density of 100,000 cells/cm^2^ in the transwell plates. According to a previous finding [40], a confluent monolayer of BeWo cells is formed for transport experiments on day 6 post-seeding. After seeding for 24 h in the transwell inserts, the cells were treated with RU486 (10 μM) and GW4064 (10 μM), with or without dexamethasone (500 nM) for 5 days, respectively, and the culture medium (0.1 ml apical compartment, 0.6 ml basolateral compartment) was replaced daily. At day 6 post-seeding, CDCA (20 μM) and taurocholic acid (TCA, 20 μM) at a concentration that has no cytotoxicity [43] were added to the upper compartments, and the samples were collected from the upper compartments after 120 min. The concentrations of the two BA samples (CDCA and TCA) were analyzed by the TBA assay kit according to the manufacturer’s instructions.

### Immunofluorescence analysis

Following 5-day treatment as described above, cells were washed three times with ice-cold PBS, fixed in 4% formaldehyde, and blocked for 30 min with 3% of BSA and 2% of fetal bovine serum in 0.2% Triton X-100/PBS. The cells were then incubated overnight at 4 °C with primary antibodies in blocking buffer, including rabbit anti-SLCO2B1 (1:50 dilutions), rabbit anti-BCRP (1:50 dilutions), rabbit anti- MRP4 (1:100 dilutions), and rabbit anti-GR (1:200 dilutions). The cells were washed with PBS and incubated with 1:100 diluted Cy3-conjugated secondary antibody (GB21303, Servicebio Inc., Wuhan, China) corresponding to anti-SLCO2B1, anti- BCRP, and anti-MRP4 or 1:200 diluted FITC-conjugated secondary antibody (GB22301, Servicebio Inc., Wuhan, China) corresponding to anti-GR for 60 min at room temperature. Nuclei were stained with 4′, 6-diamidino-2-phenylindole (DAPI) at 1:500 dilution for 5 min. The slides were washed twice with PBS. Negative controls obtained by omitting primary antibody showed negligible background fluorescence. Fluorescence images for transporters were captured using immunofluorescence microscopy (Nikon H550S, Tokyo, Japan). Quantitative fold change of transporter expression was analyzed by ImageJ version 1.44 (National Institutes of Health, Bethesda, USA). All the samples were scored blindly and independently by three investigators.

### Total RNA extract and RT-qPCR

Procedure details for total RNA extraction and RT-qPCR have been described previously [44]. Placenta tissues (50 mg) were obtained from the same position in each placenta, and cells were collected. Total RNA was isolated from rat placentas and BeWo cells using TRIzol reagent (Invitrogen, CA, USA) according to the manufacturer’s instructions. To transcribe total RNA into cDNA, single-strand cDNA was prepared from 2 μg total RNA according to the protocol of the Applied Biosystems TaqMan Reverse Transcription reagent kit. RT-qPCR was performed using the ABI Step One RT-qPCR thermal cycler (ABI Stepone, USA) in a 10-μl reaction volume. To determine the mRNA expression of *Slco2b1/SLCO2B1*, *Bcrp/BCRP*, *Slco4a1/SLCO4A1*, *Mrp4/MRP4*, *GR*, *FXR*, *Cyp7a1*, *Cyp27a1*, and *Slc27a5*, RT-qPCR was performed with SYBR Green dye. Premier 6.0 was applied to design primers, and their sequences are shown (Table 1). To quantify the gene transcripts more precisely, the mRNA level of the housekeeping gene glyceraldehyde 3-phosphate dehydrogenase (GAPDH) was measured and used as a quantitative control. Each sample was normalized against GAPDH mRNA level. Relative amplicon expression was calculated using the 2^−ΔΔCt^ method.

**Table 1:** Oligonucleotide primers and PCR conditions of rat and human in real-time quantitative PCR

Genes	Forward primer (5′-3′)	Reverse primer (3′-5′)	Annealing
**Rat**			
*GR*	CACCCATGATCCTGTCAGTC	AAAGCCTCCCTCTGCTAACC	61 °C, 30 s
*FXR*	GATGTCTTGGAGGGTGAATG	GAGTGAGACCTGGTACAAATG	60 °C, 30 s
*Oatp2b1*	TGTCTGCCGCTACTATGA	CTTGTAGGTCTGAGCTCTTTAC	60 °C, 30 s
*Bcrp*	GAGCCTTCCAAGAGAGAGA	CTGATGACAGAACGAGGTAAC	60 °C, 30 s
*Oatp4a1*	GGTGTGACAACTGGCTATG	GCATAGCAGGGAGAGTAGTA	60 °C, 30 s
*Mrp4*	GTGTTGGACAGAGACAGTTAG	TGTGAGCAATGGTGAGAAC	60 °C, 30 s
*Cyp7a1*	CCATAAGGTGTTGTGCCACGGAAA	GCCCAAATGCCTTCGCAGAAG	60 °C, 30 s
*Cyp27a11*	ACTGCACCAGTTACAGGTGCTTTACA	CCATGTCGTTCCGTACTGGGTACT	60 °C, 30 s
*Slc27a5*	GGAGATCACAAACACCTACAA	CCTTGTTGTCCAGTATGTAGAG	60 °C, 30 s
*GAPDH*	GCAAGTTCAATGGCACAG	GCCAGTAGACTCCACGACA	60 °C, 30 s
**Human**			
*GR*	GTTACACAGGCTTCAGGTATC	TGGAGTTTCCTTCCCTCTT	60 °C, 30 s
*FXR*	CCAAAGTCATCTCCTACTTCAG	CAGTGTCTTCCAAGCAGTAG	60 °C, 30 s
*OATP2B1*	CCCAGCACTCGTGTGGAATA	GCACGTTGAGTCGCAGGAT	60 °C, 30 s
*BCRP*	GTCAGAGTGTGGTTTCTGTAG	TGCTGCAAAGCCGTAAA	60 °C, 30 s
*OATP4A1*	CTGTATCCCTCAGAATCTTTCC	CATCGTAGAGTTGCCGTTAG	60 °C, 30 s
*MRP4*	GTTGGCATTGTGGGAAGA	CAGGTTCCTGAGGTATGATTG	60 °C, 30 s
*GAPDH*	GGGAAGCTCAAGGGAGATA	CTAAGAGACAAGAGGCAAGAAG	60 °C, 30 s

*GR* glucocorticoid receptor, *FXR* farnesoid X receptor, *Oatp2b1/OATP2B1* organic anion transporter polypeptide-related protein 2b1, *Bcrp/BCRP* breast cancer resistance protein, *Oatp4a1/OATP4A1* organic anion transporter polypeptide-related protein 4a1, *Mrp4/MRP4* multidrug resistance-associated proteins 4, *Cyp7a1* cytochrome P450 family 7 subfamily A member 1, *Cyp27a1* cytochrome P450 family 27 subfamily A member 1, *Slc27a5* solute carrier family 27 member 5, *GAPDH* glyceraldehyde-3-phosphate dehydrogenase

### Total protein extract and western blotting assays

Western blotting was performed as previously described [45]. Briefly, homogenate placenta tissues or cells were rinsed with ice-cold PBS and then lysed for 30 min at 4 °C in radioimmunoprecipitation assay (RIPA) lysis buffer containing phosphatase inhibitor cocktail, followed by the BCA Assay Kit for protein quantification. A total of 30 μg of proteins was loaded to each lane, isolated by SDS-PAGE (10% gels), and blotted onto polyvinylidene difluoride (PVDF) membranes (Millipore, MA, USA). Membranes were blocked in 5% non-fat milk for 1 h and incubated overnight at 4 °C with the primary antibody, including rabbit anti-GR (1:100 dilutions), mouse anti-FXR (1:500 dilutions), and mouse anti-GAPDH (1:5000 dilutions). Then, they were incubated with horseradish peroxidase (HRP)-conjugated secondary antibody (goat anti-rabbit IgG, 1:5000; goat anti-mouse IgG, 1:5000) for 1 h and visualized using electrochemiluminescence (ECL) HRP substrate (PerkinElmer, Inc., Boston, MA). Signals of antibody binding were detected by Chemi-doc Image Analyzer (Bio-Rad, Hercules, CA). Relative protein levels were standardized with the GAPDH protein level. Protein band intensities were analyzed by ImageJ (National Institutes of Health, Bethesda, MD) from three independent bands.

### Statistical analysis

The experimental data were presented as mean ± SD. Statistical analysis was conducted with SPSS 20.0 (SPSS Science Inc., Chicago, IL) and GraphPad Prism 7 (GraphPad Software Inc., San Diego, CA, USA). Comparisons between two groups were tested by Student’s *t* test, and the difference among multiple groups was evaluated by one-way analysis of variance (ANOVA) followed by Tukey’s post hoc procedure. For human samples, the Mann-Whitney *U* test was performed. Statistical significance was defined as *P* < 0.05.

## Results

### Effects of prenatal dexamethasone therapy on human neonatal serum BA levels and the expression of placental BA transporters

**Fig. 1:**
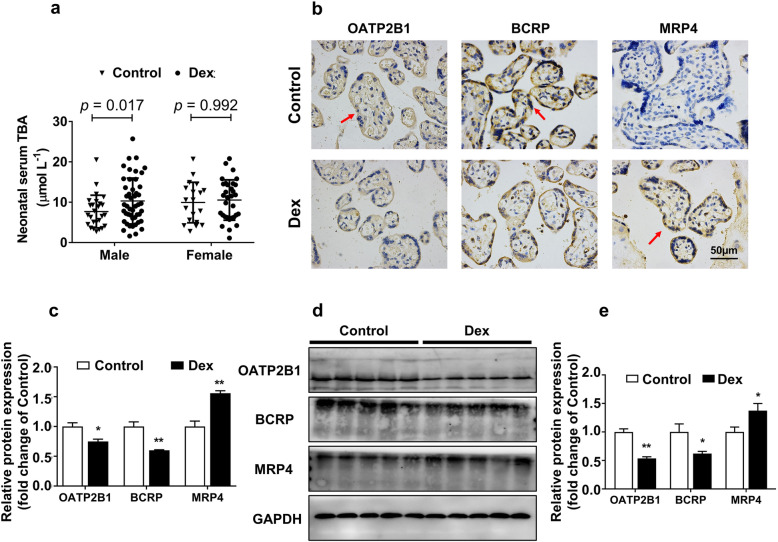
Effects of dexamethasone on human neonates’ bile acid level and placental bile acid transporters’ expression. Total bile acid (TBA) levels in neonatal serum from controls and prenatal dexamethasone-treated pregnant women (**a**). Immunohistochemistry was applied to detect the protein localization and expression of organic anion transporter polypeptide-related protein 2B1 (OATP2B1), breast cancer resistance protein (BCRP), and multidrug resistance-associated proteins 4 (MRP4) in paraffin-embedded male neonatal placenta tissue sections (5 μm) (**b**, **c**). Images were taken at × 400 magnification. Scale bars 50 μm. *n* = 5 placentas per group and three random areas in each section were scored. The protein expression of OATP2B1, BCRP, and MRP4 in male neonatal placentas (**d**, **e**) were measured by western blotting. Data are presented as mean ± SD. *P < 0.05 vs. control. **P < 0.01 vs. control. Dex, dexamethasone

We conducted a retrospective analysis of the serum BA level in male neonates who were admitted immediately to the neonatal intensive care unit from June 1 to December 1, 2017, in Zhongnan Hospital of Wuhan University. The characteristics of the enrolled subjects are shown in Table 2. In the control group, a percentage of pregnant women underwent provider-initiated preterm birth (male 37.9%, female 36.8%), which was similar to the dexamethasone-treated group (male 44.7%, female 41.4%). Besides, no significant differences were found in maternal age, gestational age, birth weight, and Apgar score at 1 and 5 min. We found that the male neonatal serum TBA level increased in the prenatal dexamethasone therapy group, while no change was found in that of the female neonatal serum (*P* < 0.05, Fig. 1a). Moreover, the male neonatal placentas were collected to detect the localization and expression of BA transporters by immunohistochemistry and western blotting, respectively. The characteristics of the enrolled subjects are shown in Table 3. We found that BA transporters, including OATP2B1, BCRP, and MRP4, were expressed in the syncytiotrophoblast of the human placentas (Fig. 1b). The protein levels of OATP2B1 and BCRP were decreased in the placentas from the prenatal dexamethasone therapy group, while MRP4 protein expression was increased (*P* < 0.05, *P* < 0.01, Fig. 1c–e). These results indicate that prenatal dexamethasone therapy can increase serum TBA level and promote the expression of placental OATP2B1 and BCRP while inhibiting MRP4 expression in the male neonates.

**Table 2:** Characteristics of enrolled male and female neonates

Characteristic	Male		*P* value	Female		*P* value
	Control *n* = 29	Dex *n* = 47		Control *n* = 19	Dex *n* = 29	
Clinical assessment of imminent preterm birth^a^						
Spontaneously initiated preterm birth	18 (62.1)	26 (55.2)		12 (63.2)	17 (58.6)	
Preterm prelabor rupture of membranes	8 (27.7)	11 (23.4)		6 (31.6)	10 (34.5)	
Spontaneous preterm labor	10 (34.5)	15 (31.9)		6 (31.6)	7 (24.1)	
Provider-initiated preterm birth	11 (37.9)	21 (44.7)		7 (36.8)	12 (41.4)	
Maternal age (years old)	32 ± 7	32 ± 6	N.S.	31 ± 7	32 ± 6	N.S.
Gestational age (weeks)	35.2 ± 0.2	34.7 ± 0.1	N.S.	35.5 ± 0.2	34.6 ± 0.2	N.S.
Birth weight (g)	2386 ± 530	2178 ± 523	N.S.	2226 ± 531	2294 ± 441	N.S.
Apgar score < 7 (5 min)^a^	2 (7)	0 (0)	N.S.	0 (0)	1 (3.4)	N.S.

^a^Values are presented as number (percentage). The rest of the data are presented as mean ± SD. *NS* no significance, *Dex* dexamethasone

**Table 3:** Characteristics of enrolled male fetal placentas

Group	Control *n* = 5	Dex *n* = 5	*P* value
Maternal age (years old)	33 ± 5	31 ± 4	NS
Gestational age (week)	35.7 ± 0.4	35.2 ± 0.4	NS
Birth weight (g)	2618 ± 277	2514 ± 284	NS
Fetal serum TBA (μmol L^−1^)	4.3 ± 1.7	12.5 ± 3.5	0.012

Data are presented as mean ± SD. *NS* no significance, *Dex* dexamethasone, *TBA* total bile acid

### Effects of PDE on TBA concentrations in rat fetal serum, placenta, and maternal serum

**Fig. 2:**
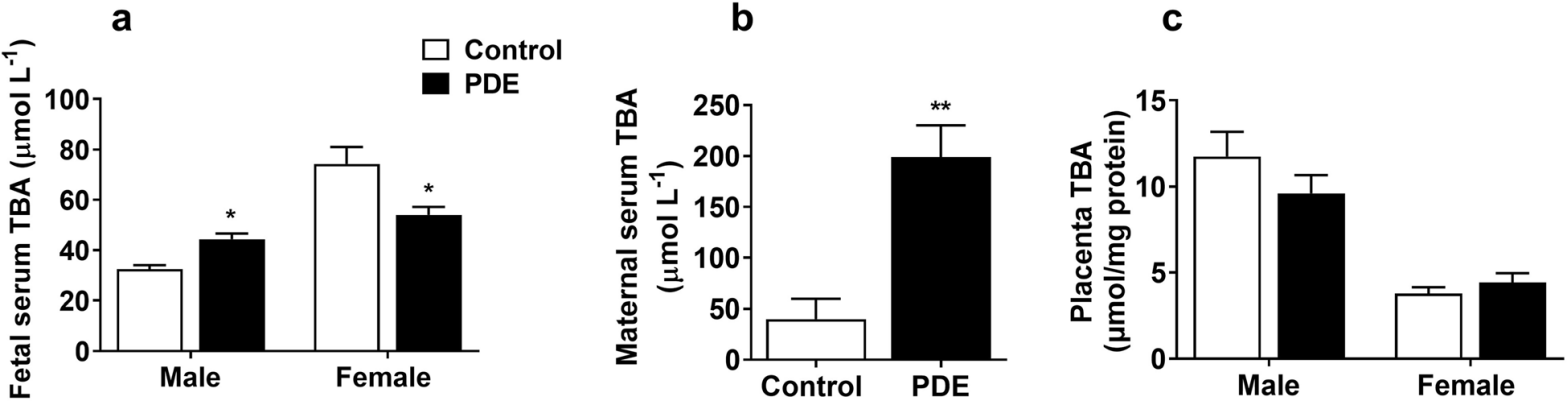
Effects of dexamethasone on bile acid levels in rat fetal serum, maternal serum, and placenta. Total bile acid (TBA) levels of male and female fetal serum (**a**), maternal serum (**b**), and placenta (**c**) were measured by TBA test kits according to the manufacturer’s protocol. Data are presented as mean ± SD, *n* = 11 litters and placentas from 11 pregnant rats in each group. *P < 0.05 vs. control. **P < 0.01 vs. control. PDE, 0.2 mg kg^−1^ day^−1^ dexamethasone

We found that, compared with the control group, male rat fetal serum TBA concentration was increased in the PDE group, while serum TBA concentration was decreased in the female PDE (*P* < 0.05, Fig. 2a). Furthermore, maternal serum TBA concentration was significantly increased in the PDE group (*P* < 0.01, Fig. 2b), while there was no significant change in rat placental TBA concentration (Fig. 2c). These results indicate that PDE can alter TBA concentrations in fetal and maternal serum, without affecting the placental TBA level in rats.

### Effects of PDE on the metabolomic spectrum of BAs in rat fetal and maternal serum

**Fig. 3:**
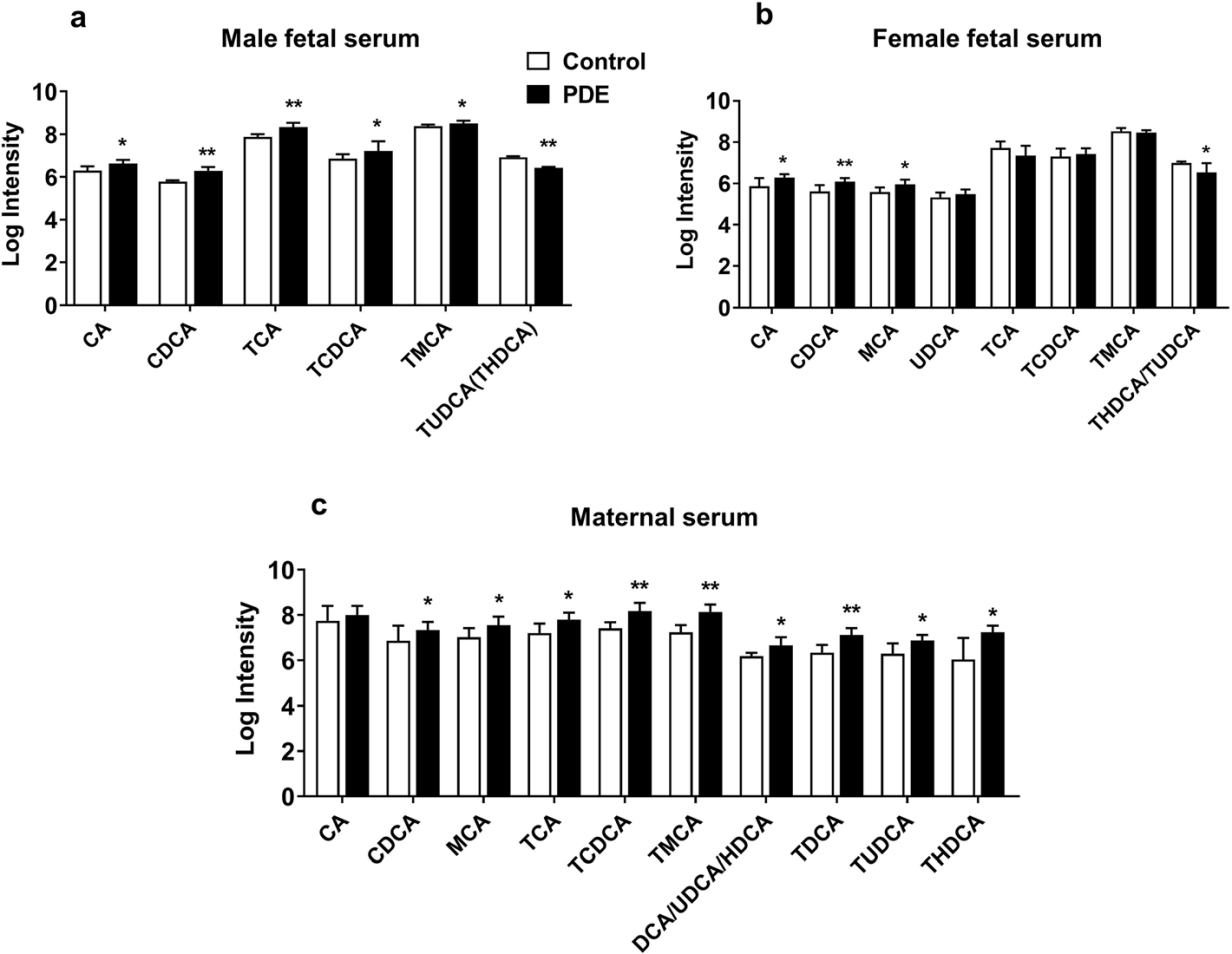
Effects of dexamethasone on rat fetal and maternal serum bile acid metabolic profile. Bile acids’ metabolic profile of male fetal serum (**a**), female fetal serum (**b**), and maternal serum (**c**) were determined by liquid chromatography/mass spectrometry (LC-MS). The relative amount of bile acid was displayed as a log intensity of the peak area. Data are presented as mean ± SD, *n* = 11 per group. *P < 0.05 vs. control. **P < 0.01 vs. control. CA, cholic acid; CDCA, chenodeoxycholic acid; MCA, muricholic acid; TCA, taurocholic acid; TCDCA, tauro-chenodeoxycholic acid; TMCA, tauro-muricholic acid; DCA, deoxycholic acid; UDCA, ursodeoxycholic acid; HDCA, hyodeoxycholic acid; TDCA, tauro-deoxycholic acid; TUDCA, tauro-ursodeoxycholic acid; THDCA, tauro-hyodeoxycholic acid; PDE, 0.2 mg kg^−1^ day^−1^ dexamethasone

LC-MS was used to analyze PDE-induced metabolomic spectrum changes of BAs in fetal and maternal serum. We found that the levels of primary unconjugated BA, including CA and CDCA, and primary conjugated BA, including TCA, tauro-chenodeoxycholic acid (TCDCA), and tauro-muricholic acid (TMCA), were increased in the male fetal serum of the PDE group (*P* < 0.05, *P* < 0.01, Fig. 3a). In the female fetal serum, the primary unconjugated BA levels, including CA, CDCA, and MCA, were increased in the PDE group (*P* < 0.05, *P* < 0.01, Fig. 3b). Besides, the levels of maternal serum BAs including primary unconjugated BAs (CDCA, MCA, and ursodeoxycholic acid), primary conjugated BAs (TCA, TCDCA, TMCA, and tauro-ursodeoxycholic acid), secondary unconjugated BAs (deoxycholic acid and hyodeoxycholic acid), and secondary conjugated BAs (tauro-deoxycholic acid and tauro-hyodeoxycholic acid) were significantly increased in the PDE group (*P* < 0.05, *P* < 0.01, Fig. 3c). These results indicate that PDE can cause abnormal metabolic profiles of BAs in fetal and maternal serum, with primary unconjugated BAs mainly increased in fetal serum. In contrast, all kinds of BA components were increased in the maternal serum.

### Effects of PDE on fetal rat hepatic BA synthesis and placental BA transport

**Fig. 4:**
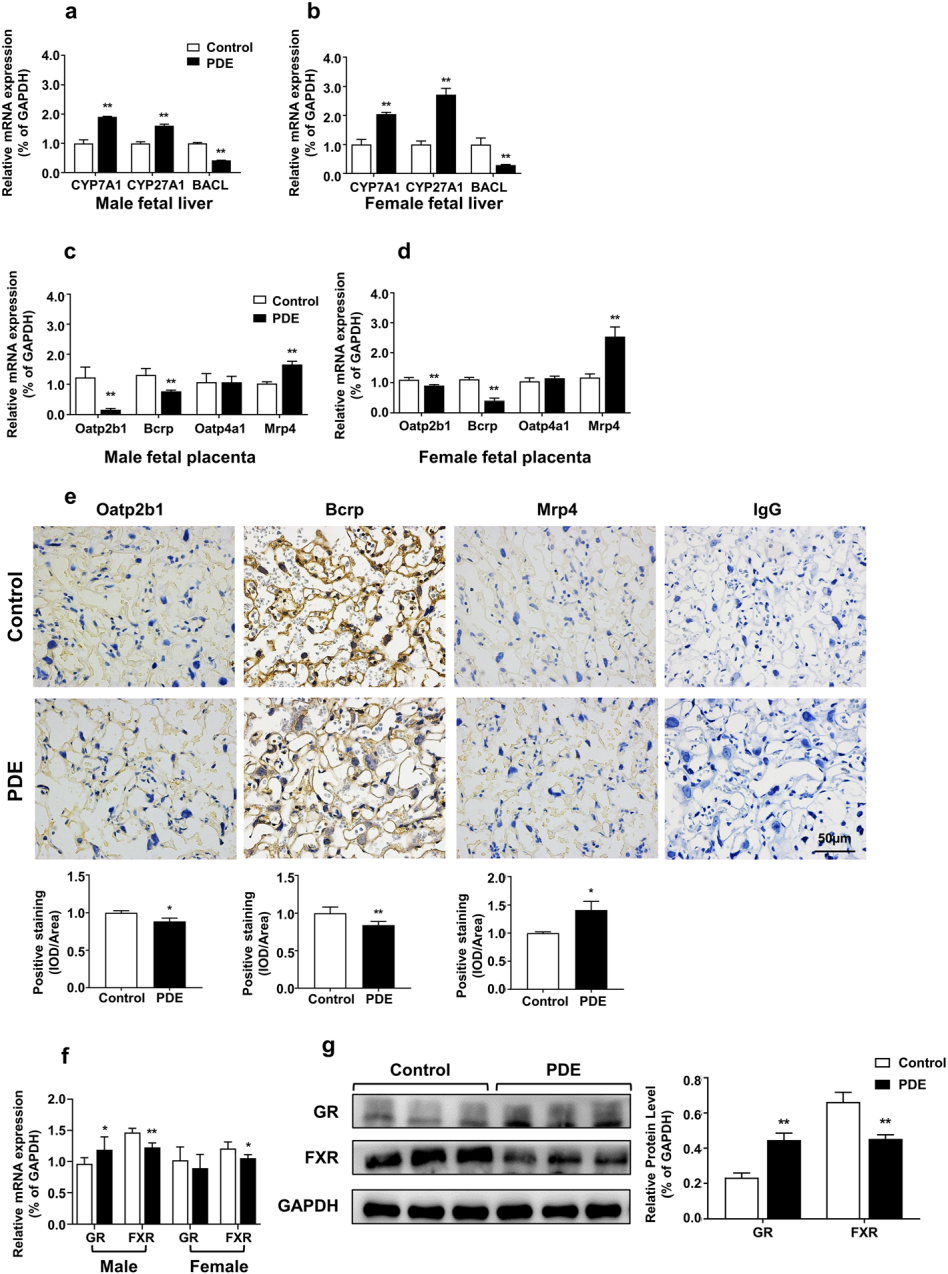
Effects of dexamethasone on the expression of fetal rat hepatic BA metabolic enzymes and rat placental bile acid transporters and nuclear receptors. The mRNA abundance of cytochrome P450 family 7 subfamily A member 1 (*Cyp7a1*), cytochrome P450 family 27 subfamily A member 1 (*Cyp27a1*), and bile acid-CoA ligase (*Bacl*) in male (**a**) and female (**b**) fetal livers was measured by RT-qPCR. The mRNA expression of organic anion transporter polypeptide-related protein 2b1 (*Oatp2b1*), breast cancer resistance protein (*Bcrp*), organic anion transporter polypeptide-related protein 4a1 (*Oatp4a1*), and multidrug resistance-associated proteins 4 (*Mrp4*) in male (**c**) and female (**d**) placentas were measured by RT-qPCR. Immunohistochemistry for Oatp2b1, Bcrp, and Mrp4 (**e**) in paraffin-embedded placenta tissue sections (5 μm) from the control group and PDE group (magnification × 400). Scale bars 50 μm. *n* = 5 placentas from five pregnant rats, and three random fields of each section were scored. The corresponding anti-IgG served as non-specific controls (**e**), and quantitative fold changes of transporters expression were analyzed by ImageJ version 1.44. The mRNA expression of the glucocorticoid receptor (GR) and farnesoid X receptor (FXR) in male and female (F) fetal placentas were measured by RT-qPCR. The protein expression of GR and FXR in male fetal placentas (**g**) were measured by western blotting. In each group, *n* = 11 livers and *n* = 11 placentas for qRT-PCR, *n* = 3 for western blotting. Data are presented as mean ± SD. *P < 0.05 vs. control. **P < 0.01 vs. control. PDE, 0.2 mg kg^−1^ day^−1^ dexamethasone

The fetal liver is able to synthesize BAs. We first detected the effects of PDE on the expression of BA metabolic enzymes in fetal rat liver. Compared with the control group, the mRNA expression levels of *Cyp7a1* and *Cyp27a1* were significantly increased in the liver of male and female fetal rats, while *Bacl* expression (encoded by *Slc27a5*) was decreased (*P* < 0.01, Fig. 4a, b). In male and female rat placentas, the mRNA expression levels of *Oatp2b1* and *Bcrp* were significantly decreased and the *Mrp4* mRNA expression was significantly increased in the PDE group (*P* < 0.01, Fig. 4c, d), while *Oatp4a1* expression did not change. Immunohistochemistry revealed that Oatp2b1, Bcrp, and Mrp4 were expressed in the placental labyrinthian region, and the protein levels of placental Oatp2b1 and Bcrp decreased. In contrast, Mrp4 protein expression increased in the male PDE group (*P* < 0.05, *P* < 0.01, Fig. 4e). Literature suggests that the expression of BA transporters is directly related to the regulation of nuclear receptors [46, 47]. We found that the mRNA and protein expression of GR was increased, while that of FXR was decreased, in male PDE placentas; in the female PDE group, only FXR mRNA expression was decreased while GR mRNA expression was not significantly changed (*P* < 0.05, *P* < 0.01, Fig. 4f, g). These results indicate that the expression of Cyp7a1 and Cyp27a1 increases, while Bacl expression decreases, in PDE fetal livers. In PDE placentas, the expression of Bcrp and Oatp2b1 was downregulated and that of Mrp4 was upregulated, while GR expression was increased and FXR expression was decreased.

### Effects of dexamethasone on the expression of BA transporters and nuclear receptors in BeWo cells

**Fig. 5:**
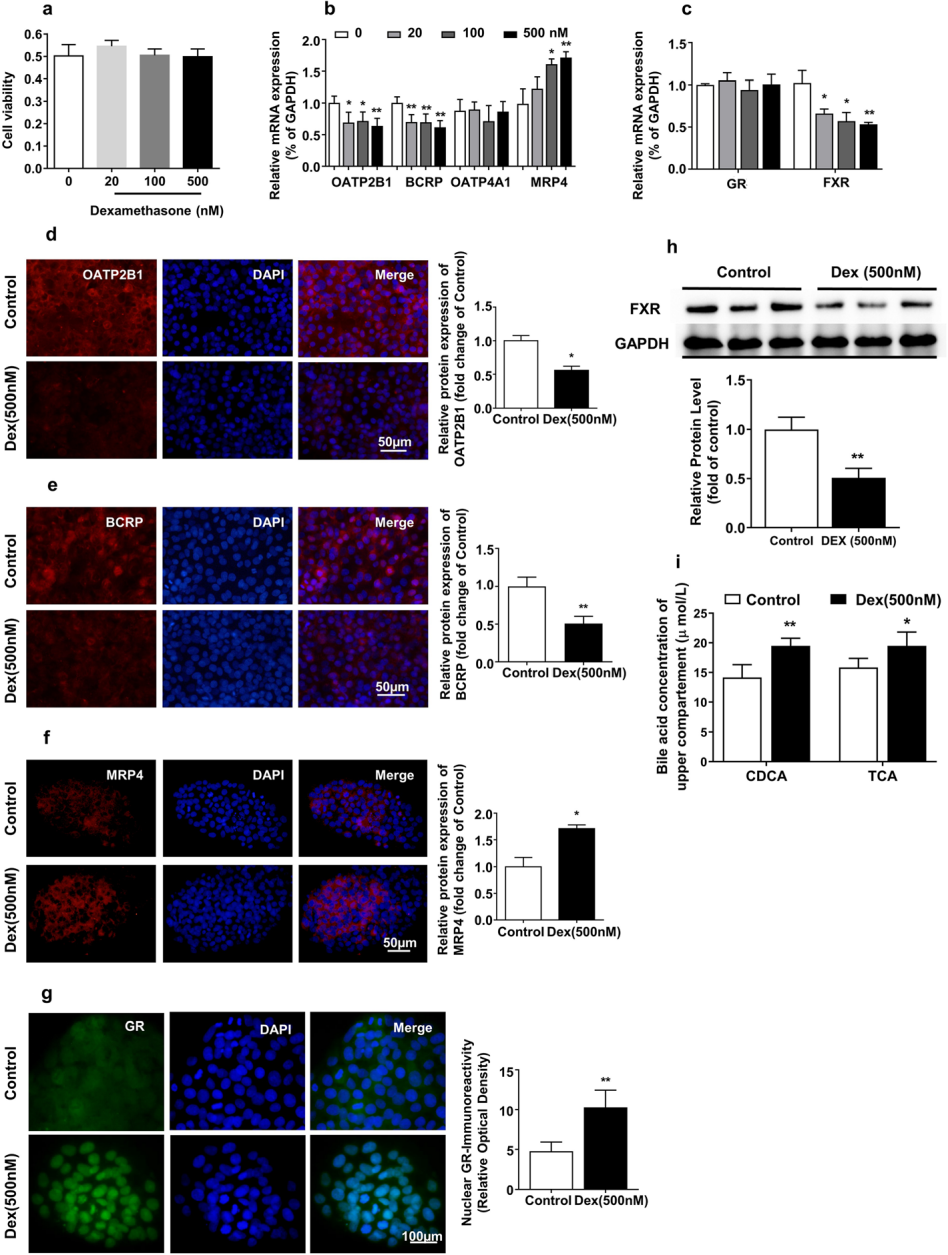
Effects of dexamethasone on expression of bile acid transporters and nuclear receptors in BeWo cells. BeWo cells were treated with dexamethasone (0, 20,100, and 500 nM) for 5 days; cytotoxicity of dexamethasone was measured by MTS assay (**a**). The mRNA expression of organic anion transporter polypeptide-related protein 2B1 (*OATP2B1*), breast cancer resistance protein (*BCRP*), organic anion transporter polypeptide-related protein 4A1 (*OATP4A1*), and multidrug resistance-associated proteins 4 (*MRP4*) (**b**), glucocorticoid receptor (*GR*), and farnesoid X receptor (*FXR*) (**c**) were measured by RT-qPCR. Cells were treated with 500 nM dexamethasone for 5 days; the protein expression of bile acid transporters (OATP2B1, BCRP, and MRP4) (**d**–**f**) and GR protein in cytoblast (**g**) were detected by immunofluorescence (magnification × 400, except “H” × 200). The protein expression of FXR was measured by western blotting, and quantitative fold change of protein level was analyzed by ImageJ version 1.44 (**h**). Cells were treated with 500 nM dexamethasone for 5 days; after that, CDCA (20 μM) and TCA (20 μM) were added into the upper compartment of a transwell insert, and the concentration of CDCA and TCA (**i**) in the upper compartment was measured by TBA test kit after 120-min incubation at 37 °C. *n* = 11 for maternal serum detection, *n* = 6 for the rest of the experiments. Data are presented as mean ± SD. *P < 0.05 vs. control. **P < 0.01 vs. control. Dex, dexamethasone

To confirm the direct effects of dexamethasone on the expression of placental BA transporters and nuclear receptors, BeWo cells were treated with 20, 100, and 500 nM dexamethasone for 5 days. The MTS assay showed that dexamethasone in the selected concentration range had no cytotoxicity on BeWo cells (Fig. 5a). We found that dexamethasone decreased *OATP2B1* and *BCRP* mRNA expression and increased the *MRP4* mRNA expression in a concentration-dependent manner (*P* < 0.05, *P* < 0.01, Fig. 5b), while no change was observed in *OATP4A1* expression. Moreover, dexamethasone decreased *FXR* mRNA expression in a dose-dependent manner (*P* < 0.05, *P* < 0.01, Fig. 5c), while it did not affect *GR* mRNA expression. Immunofluorescence showed that dexamethasone (500 nM) decreased OATP2B1and BCRP protein expression, increased MRP4 expression (*P* < 0.05, *P* < 0.01, Fig. 5d–f), and induced GR translocation into the nucleus (*P* < 0.01, Fig. 5g). Besides, dexamethasone (500 nM) reduced FXR protein expression (*P* < 0.01, Fig. 5h). TCA is known as the predominant form among the primary conjugated BAs [48], and we found that CDCA as a main component of the primary unconjugated BAs was greatly increased in male fetal serum. Therefore, we chose TCA and CDCA as the substrates to detect the effect of dexamethasone on placental BA transport activity. CDCA and TCA concentrations in dexamethasone (500 nM)-treated upper compartments were higher than those in the control (*P* < 0.05, *P* < 0.01, Fig. 5i). These results indicate that dexamethasone can activate GR and decrease FXR expression, thus inhibiting OATP2B1 and BCRP expression and enhancing MRP4 expression, eventually decreasing BA transport activity.

### GR- and FXR-mediated dexamethasone-induced expression changes of various BA transporters in BeWo cells

**Fig. 6:**
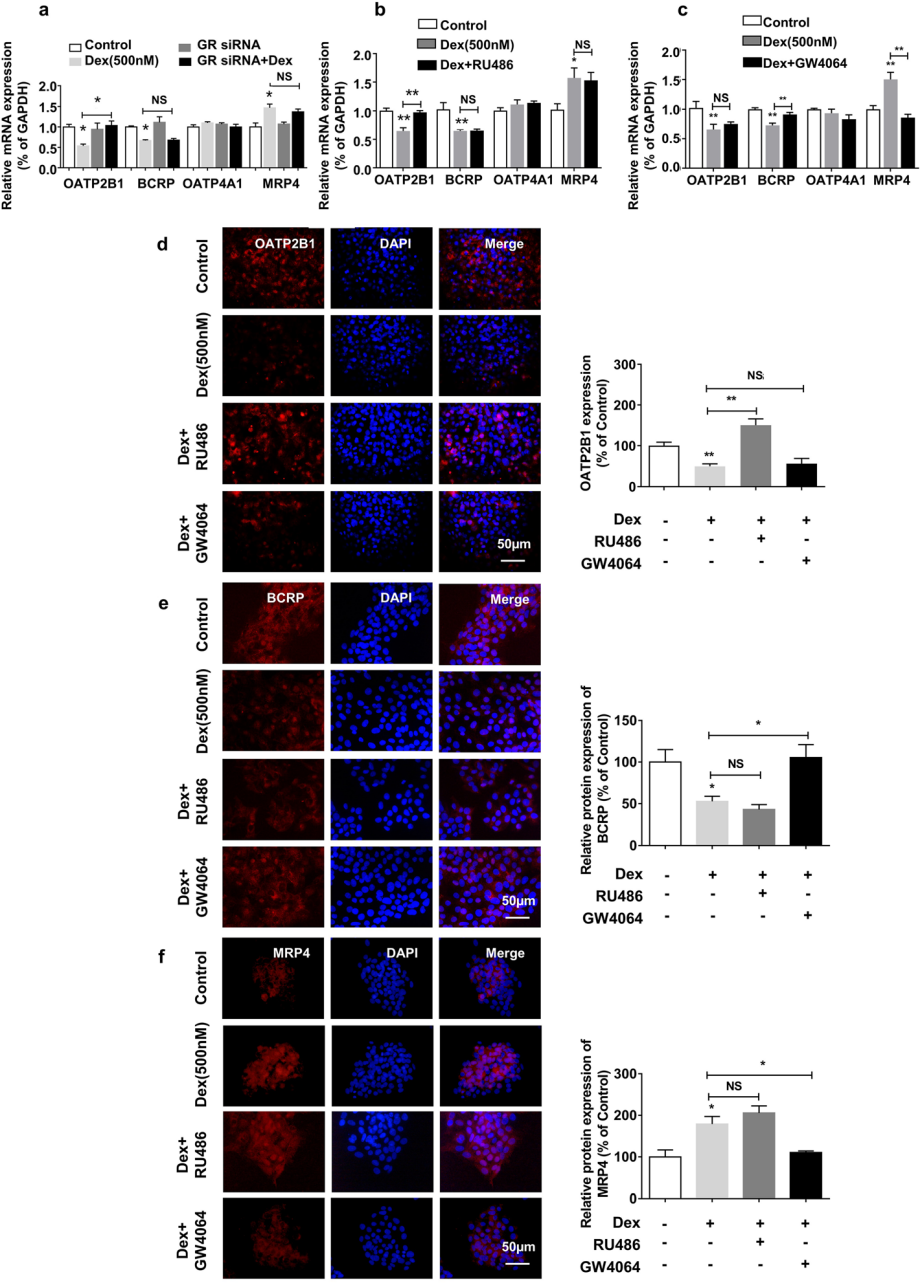
Effects of GR siRNA, RU486, and GW4064 on the expression of bile acid transporters in the human BeWo cells. BeWo cells were transiently transfected with 100 nM GR siRNA, 100 nM negative control (i.e., scrambled) siRNA. After 24-h transfection, cells were treated with or without 500 nM dexamethasone for 3 days. At the end of treatment, mRNA expression of organic anion transporter polypeptide-related protein 2B1 (*OATP2B1*), breast cancer resistance protein (*BCRP*), organic anion transporter polypeptide-related protein 4A1 (*OATP4A1*), and multidrug resistance-associated proteins 4 (*MRP4*) (**a**) were measured by RT-qPCR. BeWo cells were treated with 500 nM dexamethasone, 500 nM dexamethasone plus 10 μM RU486, or 500 nM dexamethasone plus GW4064 for 5 days. At the end of treatment, mRNA expression of OATP2B1, ABCG2, and ABCC4 (**b**, **c**) were measured by RT-qPCR, and the protein expression of OATP2B1, BCRP, and MRP4 (**d**–**f**) were measured by immunofluorescence staining (magnification × 400). Scale bars 50 μm. Quantitative fold changes of transporter expression were analyzed by using ImageJ version 1.44. *n* = 6 per group. Data are presented as mean ± SD. *P < 0.05 vs. control. **P < 0.01 vs. control. Dex, dexamethasone; NS, no significance

In order to confirm that GR and FXR are involved in dexamethasone-induced expression changes of various placental BA transporters, we treated BeWo cells with GR siRNA, RU486 (a GR antagonist), or GW4064 (an FXR agonist). GR siRNA or RU486 blocked the inhibitory effect of dexamethasone on *OATP2B1* mRNA expression, while it had no effect on *BCRP* and *MRP4* expression (*P* < 0.05, *P* < 0.01, Fig. 6a, b). GW4064 abrogated dexamethasone-induced downregulation of *BCRP* and upregulation of *MRP4*, while it had no effect on *OATP2B1* expression (*P* < 0.01, Fig. 6c). The similar effects of RU486 and GW4064 on the expression of transporters were also observed at the protein level by immunofluorescence (*P* < 0.05, *P* < 0.01, Fig. 6d–f). Taken together, these results indicate that GR mediates OATP2B1 downregulation induced by dexamethasone in placental trophoblast cells, while FXR mediates BCRP downregulation and MRP4 upregulation.

## Discussion

### PDE alters TBA levels and BA profiles in human and rat fetal serum

Dexamethasone has been widely used for pregnant women at risk of preterm delivery between 24 and 34 weeks of gestation. The World Health Organization (WHO) recommended in 2015 the use of antenatal glucocorticoids only under certain conditions, including the accurate assessment of the gestational age, imminent preterm labor, the absence of maternal infection, and sufficient care for childbirth and preterm newborns [49]. Recent studies demonstrated that the appropriate use of antenatal dexamethasone in low-resource countries can reduce the neonatal mortality and stillbirth [50, 51]. The classic therapeutic use of dexamethasone in the clinic is 6 mg intramuscular injection per 12 h, and four times for one course of treatment. However, due to the difficulty in early diagnosis of precise delivery timing and the possible failure of single-course dexamethasone treatment, the ideal window for corticosteroid delivery cannot always be reached [52], and a rescue course of dexamethasone has been administered in some undelivered women who are still at risk of preterm birth after 7 days of the first course [53]. Moreover, the use of prenatal dexamethasone therapy has a double-edged sword effect. Dexamethasone can promote fetal lung maturation and effectively reduce premature infant mortality [54] but can also induce intrauterine growth retardation and developmental toxicity and related diseases in various organs in adult offspring [26–28]. These findings highlighted the importance of the appropriate use of antenatal dexamethasone to achieve the greatest benefits.

Previous studies demonstrated that accumulated hydrophobic BAs lead to significant oxidative damage and apoptosis in the placenta and fetal liver [3, 55]. Our previous studies found that dexamethasone can induce maternal intrahepatic cholestasis [31]. Moreover, their adult offspring also exhibited cholestatic liver injury [30]. These findings suggested that PDE can lead to abnormal BA metabolism and increase the susceptibility to cholestatic liver injury in the mother and offspring. However, little is known about the effects of prenatal dexamethasone on fetal BA metabolism.

In this clinical study, we found that serum TBA level significantly increased in male neonates whose mothers received prenatal dexamethasone therapy at 24–34 gestational weeks but failed to deliver at the target window, or those who were treated with dexamethasone at 34–37 gestational weeks. Besides, in our rat model, PDE was confirmed to increase male fetal and maternal serum TBA levels. Furthermore, LC-MS analysis demonstrated that primary BAs were significantly increased in both male and female fetal serum, while all BA components were significantly increased in the maternal serum. These results suggested that PDE could increase hydrophobic BA levels in both fetal and maternal serum, which may be associated with imbalanced fetal-placental-maternal BA circulation during pregnancy.

### PDE-induced imbalance of fetal-placental-maternal BA circulation is mainly mediated by placental transporters’ alteration

The fetal liver can synthesize primary unconjugated BAs in early life through classical and alternative pathways. However, to our knowledge, there is no published article about the effects of PDE on the expression of bile acid metabolic enzymes in the fetal liver. Only one study showed that dexamethasone treatment at 1–3 days after birth significantly increased the expression of Cyp7a1 and Cyp27a1 in the liver of rat offspring [56]. Dexamethasone can pass through the placenta to reach the fetus [57]. Therefore, we detected the expression of BA metabolic enzymes in the fetal rat livers and found that PDE increased Cyp27a1 and Cyp7a1 expression while decreasing Bacl expression, speculating that PDE may promote the synthesis of unconjugated BAs in the fetal rat liver while inhibiting the synthesis of conjugated BAs. However, we found that the levels of primary conjugated BAs (including TCA, TCDCA, and TMCA) in male fetal rat serum were significantly increased, which cannot be fully explained by the altered fetal hepatic BA metabolic enzymes. Taken together, dexamethasone-induced fetal rat hepatic BA synthesis may not be the major factor causing the accumulation of primary conjugated BAs in fetal rat blood.

Due to the incomplete liver development and the insufficiency of intestinal flora, the intestinal-hepatic circulation is absent in the fetus. The primary BAs synthesized by the fetal liver must be transported to the mother through the placenta for further metabolism to avoid the accumulation of hydrophobic BAs in the fetus [14]. Maternal blood BAs can also be transported to the fetus [12]. Therefore, the placenta plays an important role in maintaining the balance of fetal-placental-maternal BA circulation. In this study, we collected human placental tissues and found that prenatal dexamethasone therapy decreased the expression of OATP2B1 and BCRP in male neonatal placenta and increased MRP4 expression. Furthermore, PDE decreased the expression of Oatp2b1 and Bcrp in the rat placental tissue. In BeWo cells, dexamethasone inhibited the expression of OATP2B1 and BCRP and reduced BA transport through the trophoblast cells. These findings suggested that dexamethasone could block the transport of fetal BAs to the mother by inhibiting the expression of placental OATP2B1 and BCRP, ultimately causing the accumulation of hydrophobic primary BAs in fetal blood. Studies have demonstrated that MRP4 expression is much higher in the placenta than in the liver [58], and MPR4 can favor BA flow from the mother to the fetus under the maternal cholestatic condition [11, 59]. In this study, all BA components in the maternal rat serum were significantly increased; the in vivo and in vitro experiments also revealed that dexamethasone increased the expression of MRP4 in the placental trophoblast cells, which indicates that MRP4 can promote the transport of maternal BAs to the fetus and further aggravate the accumulation of primary conjugated BAs in the fetal blood. Therefore, the increased primary conjugated BAs in male fetal blood may be mainly attributed to the increase of maternal primary conjugated BA level and the enhancement of placental MRP4 expression. All these findings suggested that dexamethasone-induced placental BA transport changes play a crucial role in the imbalance of fetal-placental-maternal BA circulation.

Interestingly, in the early days, dexamethasone has been applied clinically to treat the intrahepatic cholestasis of pregnancy [60]. However, our recent study found that PDE increased the levels of TBA and all BA components in maternal rat serum, leading to intrahepatic cholestasis of the mother [31], suggesting that the disadvantages of long-term dexamethasone use during pregnancy outweighed the advantages to maternal BA metabolism. Our previous study also showed that PDE-induced upregulation of BA synthetic enzymes (e.g., Cyp7a1) and downregulation of various BA transporters in the liver were responsible for the changes of BAs in maternal serum [31]. In this study, all BA components in maternal serum were significantly increased, which was distinct from fetal BA metabolic profile, indicating that the maternal BA metabolism is independent of the fetus.

### Dexamethasone regulates the expression of placental BA transporters through GR and FXR nuclear receptors

An increasing number of studies have demonstrated that dexamethasone can alter the expression of hepatic BA transporters through some nuclear receptors, such as GR and FXR, resulting in hepatic cholestasis [20, 21]. Investigators have found that the hepatic regulation of BA transporters involves a complex network of nuclear receptors, among which FXR is considered as the most important BA sensor [61–63]. Although an interaction between dexamethasone and FXR has been observed [20], dexamethasone also antagonizes the expression of genes involved in the hepatic handling of BAs through FXR-independent mechanisms [21]. In addition, dexamethasone regulates the expression of BA transporters in a tissue-specific way. For example, dexamethasone increases BCRP expression in rat brain endothelial cells [64] but reduces BCRP expression in placental trophoblast cells [65]. Previous studies have shown the expression of GR and FXR in the placenta [22, 23]. In this study, we confirmed that dexamethasone can inhibit the expression of OATP2B1 and BCRP, while upregulating MRP4 expression in the placental trophoblast cells. At the same time, we further found that GR antagonist or GR siRNA only blocked dexamethasone-induced decrease of OATP2B1 expression, indicating that dexamethasone-activated GR is involved in the downregulation of OATP2B1. Using the FXR agonist, we also verified that FXR mediates dexamethasone-induced BCRP and MRP4 expression changes. In conclusion, dexamethasone mediates OATP2B1 downregulation by activating GR, as well as BCRP downregulation and MRP4 upregulation by inhibiting FXR.

### Gender differences in the PDE-induced imbalance of fetal-placental-maternal BA circulation and its possible placental mechanism

In the field of metabolic diseases, male rodents often exhibit more pronounced disease phenotypes than their female counterpart [66, 67]. The summary of the gender differences observed in this study is shown in Table 4. In the animal study, we found that the TBA level in male fetal blood of the PDE group was significantly increased, while that in female fetal blood was decreased. In the human study, the male neonatal blood TBA level increased in the prenatal dexamethasone therapy group, while no significant change in that of female neonatal blood was observed. We further found that both primary unconjugated and conjugated BA levels were increased in male fetal blood, but only primary unconjugated BA levels were increased in female rat fetal blood. These findings suggested that fetal blood TBA levels and metabolomic spectrum of BAs exhibited significant gender differences after prenatal dexamethasone treatment, in which the male neonates (or rat fetus) showed more severe alterations than the females.

**Table 4:** Gender differences in prenatal dexamethasone exposure-induced the imbalance of fetal-placental-maternal bile acids circulation

	Male	Female
**TBA level**	Up*	Down*
**Metabolomic spectrum of BAs**		
TCA	Up**	**N. S**
TCDCA	Up*	**N. S**
TMCA	Up*	**N. S**
**Placental BA transporter**		
Oatp2b1	Down**	Down*
**Placental nuclear receptor**		
GR	Up*	**N. S**

*P < 0.05, **P < 0.01 vs control. *N. S* no significant change, *TBA* total bile acid, *BAs* bile acids, *TCA* taurocholic acid, *TCDCA* tauro-chenodeoxycholic acid, *TMCA* tauro-muricholic acid, *Oatp2b1* organic anion transporter polypeptide-related protein 2b1, *GR* glucocorticoid receptor

Preliminary experiments were conducted to investigate the possible mechanism of the gender differences in PDE-induced BA metabolic alterations. Regarding the placental transporters, we found that although the alterations of Bcrp and Mrp4 level in female placentas were consistent with those in the males, the decrease in Oatp2b1expression was not as significant as that in the males. Regarding the placental nuclear receptors, although PDE decreased FXR expression in the female placenta, there was no significant change in GR expression (PDE increased GR expression in the male placentas). Besides, we detected and compared the gene expression of hepatic BA metabolic enzymes and found that PDE increased the expression of Cyp27a1 and Cyp7a1 and decreased Bacl expression in male and female fetal livers, with no gender difference observed. These findings suggested that BA transport via the placenta, and not BA synthesis via the fetal liver, mainly contributes to the gender differences in PDE-induced BA metabolic alterations. Studies have reported that GR subtype expression profiles are different between male and female rodent placentas, and placental GR responds to dexamethasone in a gender-specific way [22]. In this study, we confirmed that dexamethasone can regulate the placental OATP2B1 expression via GR. Taken together, PDE-induced gender different patterns in the expression of GR and Oatp2b1 may be the main reason for the gender differences in placental BA transport, which may further lead to more severe alterations in male BA metabolism. However, the specific mechanism of this gender difference requires further investigation.

## Conclusion

A previous study in our lab demonstrated that dexamethasone induces mouse fetal developmental toxicity in a course-, dose-, and stage-dependent manner: multiple courses, a high dose, or the use at an early developmental stage induces stronger toxic effects on fetal development [68]. In the pregnant rats treated with dexamethasone (0.2 mg/kg per day) during the second and third trimesters, we confirmed that PDE can induce developmental toxicities in multiple organs, such as bone, hippocampus, and ovary [35, 69, 70]. In this study, we used the same PDE rat model to investigate the effects of PDE on BA metabolism in fetal rats and elucidate the underlying mechanism. Our study confirmed that PDE can increase serum BA level and induce placental function damage in fetal rats, which was consistent with the clinical changes in newborns treated with antepartum dexamethasone. It should be pointed out that although the dose of dexamethasone in the PDE rat model is achievable in the clinical practice, the duration of dexamethasone exposure is longer than the standard use in human clinical practice, which was the deficiency of this PDE rat model. Based on the consistency of the results from animal experiments and clinical studies in this study, this PDE rat model can still provide insights into the adverse effect of prenatal dexamethasone use on the fetal-placental-maternal BA circulation. In addition, we found that PDE significantly increased all kinds of BA components (primary and secondary BAs) in the maternal serum. Although the PDE rat model and human trophoblast cell line (BeWo) confirmed that the increase of primary BA in fetal blood was mainly due to the placental damage caused by dexamethasone, we cannot rule out the possible impairments of maternal cholestasis on the placenta and fetus. This is a limitation of our study and this possibility requires further studies in the future.

**Fig. 7:**
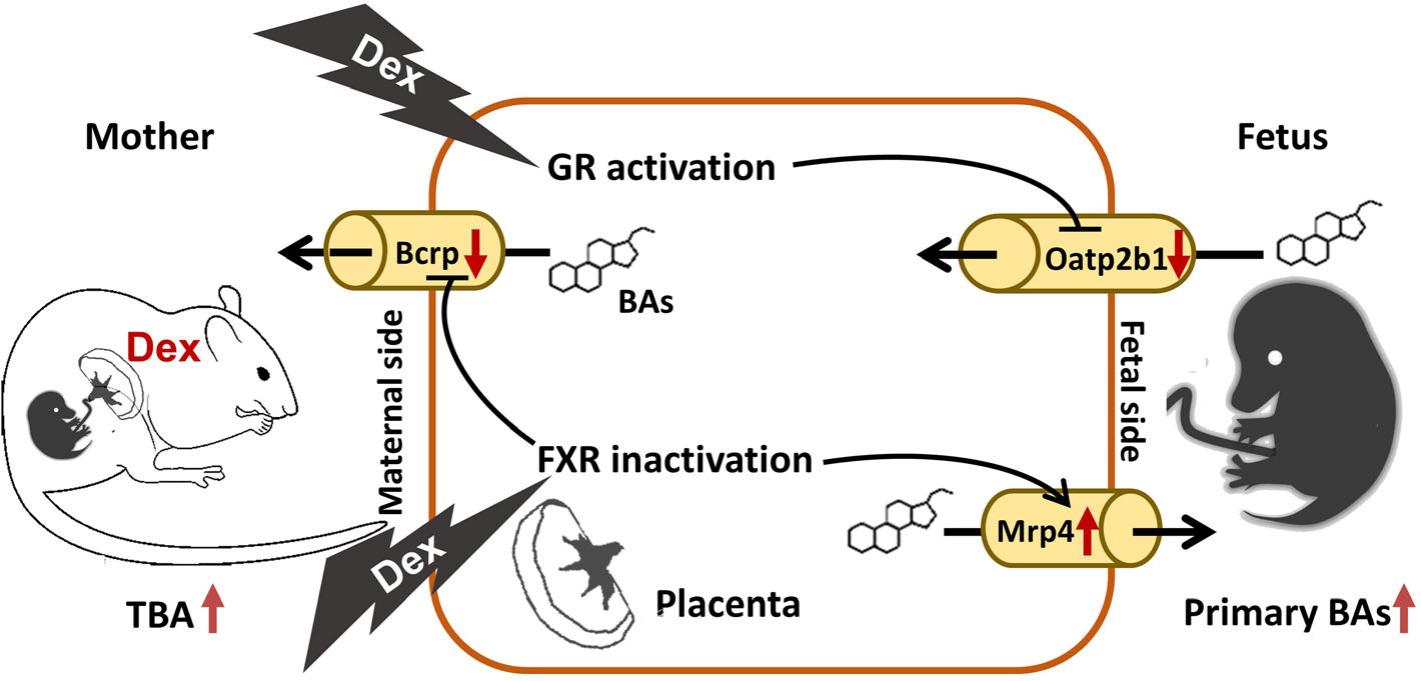
Placental mechanism in the metabolic changes of fetal serum bile acids induced by prenatal dexamethasone. BAs, bile acids; TBA, total bile acid; GR, glucocorticoid receptor; FXR, farnesoid X receptor; Bcrp, breast cancer resistant protein; Oatp2b1, organic anion-transporting polypeptides 2b1; Mrp4, multidrug resistance protein

In summary, we integrated for the first time human, animal, and cell experiments and provided evidence that PDE can elevate serum TBA level in male fetuses and affect metabolic BA profile, which was characterized by the elevation of primary unconjugated and conjugated BA levels. The potential mechanism (Fig. 7) might be that dexamethasone downregulates placental OATP2B1 via activating GR and decreases BCRP expression and enhances MRP4 expression by inhibiting FXR expression. This placental dysfunction leads to the imbalance of fetal-placental-maternal BA circulation and the accumulation of hydrophobic BAs in the fetal serum. We also found for the first time that there are some gender differences in the changes in fetal serum BA levels induced by PDE, but the exact mechanism remains to be uncovered. This study provides an important experimental and theoretical basis for elucidating the mechanism of PDE-induced maternal and offspring abnormal BA metabolism-associated diseases and for exploring early prevention and treatment strategies.

## Data Availability

The datasets used and/or analyzed during the current study are available from the corresponding author on reasonable request.

## Abbreviations

BACL: Bile acid-CoA ligase
BAs: Bile acids
BCRP: Breast cancer resistance protein
CA: Cholic acid
CDCA: Chenodeoxycholic acid
CYP27A1: Cytochrome P450 family 27 subfamily A member 1
CYP7A1: Cytochrome P450 family 7 subfamily A member 1
DCA: Deoxycholic acid
FXR: Farnesoid X receptor
GR: Glucocorticoid receptor
HDCA: Hyodeoxycholic acid
MCA: Muricholic acid
MRP4: Multidrug resistance-associated protein 4
OATP2B1: Organic anion transporter polypeptide-related protein 2B1
PDE: Prenatal dexamethasone exposure
TBA: Total bile acid
TCA: Taurocholic acid
TCDCA: Tauro-chenodeoxycholic acid
TDCA: Tauro-deoxycholic acid
THDCA: Tauro-hyodeoxycholic acid
TMCA: Tauro-muricholic acid
TUDCA: Tauro-ursodeoxycholic acid
UDCA: Ursodeoxycholic acid

## References

[1] Monte MJ, Marin JJ, Antelo A, Vazquez-Tato J (2009). Bile acids: chemistry, physiology, and pathophysiology. World J Gastroenterol.

[2] Sigurdsson V, Takei H, Soboleva S, Radulovic V, Galeev R, Siva K, Leeb-Lundberg LMF, Iida T, Nittono H, Miharada K (2016). Bile acids protect expanding hematopoietic stem cells from unfolded protein stress in fetal liver. Cell Stem Cell.

[3] Perez MJ, Macias RI, Marin JJ (2006). Maternal cholestasis induces placental oxidative stress and apoptosis. Protective effect of ursodeoxycholic acid. Placenta..

[4] Perez MJ, Macias RI, Duran C, Monte MJ, Gonzalez-Buitrago JM, Marin JJ (2005). Oxidative stress and apoptosis in fetal rat liver induced by maternal cholestasis. Protective effect of ursodeoxycholic acid. J Hepatol.

[5] Papacleovoulou G, Abu-Hayyeh S, Nikolopoulou E, Briz O, Owen BM, Nikolova V, Ovadia C, Huang X, Vaarasmaki M, Baumann M, Jansen E, Albrecht C, Jarvelin MR, Marin JJG, Knisely AS, Williamson C (2013). Maternal cholestasis during pregnancy programs metabolic disease in offspring. J Clin Invest.

[6] Memon N, Griffin IJ, Lee CW, Herdt A, Weinberger BI, Hegyi T, Carayannopoulos MO, Aleksunes LM, Guo GL (2020). Developmental regulation of the gut-liver (FGF19-CYP7A1) axis in neonates. J Matern Fetal Neonatal Med.

[7] Nakagawa M, Setchell KD (1990). Bile acid metabolism in early life: studies of amniotic fluid. J Lipid Res.

[8] Collado MC, Rautava S, Aakko J, Isolauri E, Salminen S (2016). Human gut colonisation may be initiated in utero by distinct microbial communities in the placenta and amniotic fluid. Sci Rep.

[9] Perez-Muñoz ME, Arrieta MC, Ramer-Tait AE, Walter J (2017). A critical assessment of the “sterile womb” and “in utero colonization” hypotheses: implications for research on the pioneer infant microbiome. Microbiome..

[10] Floch MH (2002). Bile salts, intestinal microflora and enterohepatic circulation. Dig Liver Dis.

[11] Marin JJ, Macias RI, Briz O, Perez MJ, Blazquez AG, Arrese M (2008). Molecular bases of the fetal liver-placenta-maternal liver excretory pathway for cholephilic compounds. Liver Int.

[12] Hassan AS, Subbiah MT (1980). Bile acids in the fetal rat: effect of maternal bile duct ligation. Steroids..

[13] Blazquez AG, Briz O, Gonzalez-Sanchez E, Perez MJ, Ghanem CI, Marin JJ (2014). The effect of acetaminophen on the expression of BCRP in trophoblast cells impairs the placental barrier to bile acids during maternal cholestasis. Toxicol Appl Pharmacol.

[14] Marin JJ, Macias RI, Serrano MA (2003). The hepatobiliary-like excretory function of the placenta. A review. Placenta.

[15] Klaassen CD, Aleksunes LM (2010). Xenobiotic, bile acid, and cholesterol transporters: function and regulation. Pharmacol Rev.

[16] Svoboda M, Riha J, Wlcek K, Jaeger W, Thalhammer T (2011). Organic anion transporting polypeptides (OATPs): regulation of expression and function. Curr Drug Metab.

[17] St-Pierre MV, Hagenbuch B, Ugele B, Meier PJ, Stallmach T (2002). Characterization of an organic anion-transporting polypeptide (OATP-B) in human placenta. J Clin Endocrinol Metab.

[18] St-Pierre MV, Stallmach T, Freimoser Grundschober A, Dufour JF, Serrano MA, Marin JJ (2004). Temporal expression profiles of organic anion transport proteins in placenta and fetal liver of the rat. Am J Physiol Regul Integr Comp Physiol.

[19] Blazquez AG, Briz O, Romero MR, Rosales R, Monte MJ, Vaquero J, Macias RIR, Cassio D, Marin JJG (2012). Characterization of the role of ABCG2 as a bile acid transporter in liver and placenta. Mol Pharmacol.

[20] Lu Y, Zhang Z, Xiong X, Wang X, Li J, Shi G (2012). Glucocorticoids promote hepatic cholestasis in mice by inhibiting the transcriptional activity of the farnesoid X receptor. Gastroenterology.

[21] Rosales R, Romero MR, Vaquero J, Monte MJ, Requena P, Martinez-Augustin O, Sanchez de Medina F, Marin JJG (2013). FXR-dependent and -independent interaction of glucocorticoids with the regulatory pathways involved in the control of bile acid handling by the liver. Biochem Pharmacol.

[22] Cuffe JSM, Saif Z, Perkins AV, Moritz KM, Clifton VL (2017). Dexamethasone and sex regulate placental glucocorticoid receptor isoforms in mice. J Endocrinol.

[23] Wu WB, Xu YY, Cheng WW, Wang YX, Liu Y, Huang D, Zhang HJ (2015). Agonist of farnesoid X receptor protects against bile acid induced damage and oxidative stress in mouse placenta--a study on maternal cholestasis model. Placenta..

[24] Vogel JP, Souza JP, Gulmezoglu AM, Mori R, Lumbiganon P, Qureshi Z (2014). Use of antenatal corticosteroids and tocolytic drugs in preterm births in 29 countries: an analysis of the WHO Multicountry Survey on Maternal and Newborn Health. Lancet (London).

[25] Bloom SL, Sheffield JS, McIntire DD, Leveno KJ (2001). Antenatal dexamethasone and decreased birth weight. Obstet Gynecol.

[26] Long NM, Shasa DR, Ford SP, Nathanielsz PW (2012). Growth and insulin dynamics in two generations of female offspring of mothers receiving a single course of synthetic glucocorticoids. Am J Obstetr Gynecol.

[27] Bensley JG, De Matteo R, Harding R, Black MJ (2012). Preterm birth with antenatal corticosteroid administration has injurious and persistent effects on the structure and composition of the aorta and pulmonary artery. Pediatr Res.

[28] Velisek L (2011). Prenatal corticosteroid exposure alters early developmental seizures and behavior. Epilepsy Res.

[29] Watkins JB, Szczepanik P, Gould JB, Klein P, Lester R (1975). Bile salt metabolism in the human premature infant. Preliminary observations of pool size and synthesis rate following prenatal administration of dexamethasone and phenobarbital. Gastroenterology..

[30] Chen G, Xiao H, Zhang J, Zhang H, Li B, Jiang T, Wen Y, Jiang Y, Fu K, Xu D, Guo Y, Ao Y, Bi H, Wang H (2019). Prenatal dexamethasone exposure-induced a gender-difference and sustainable multi-organ damage in offspring rats via serum metabolic profile analysis. Toxicol Lett.

[31] Fang M, Zhang Q, Yu P, Ge C, Guo J, Zhang Y, Wang H (2020). The effects, underlying mechanism and interactions of dexamethasone exposure during pregnancy on maternal bile acid metabolism. Toxicol Lett.

[32] Siddiqui A, Cuttini M, Wood R, Velebil P, Delnord M, Zile I, Barros H, Gissler M, Hindori-Mohangoo AD, Blondel B, Zeitlin J, the Euro-Peristat Scientific Committee (2017). Can the Apgar score be used for international comparisons of newborn health?. Paediatr Perinat Epidemiol.

[33] Gao Y, Xue Q, Chen G, Stone P, Zhao M, Chen Q (2013). An analysis of the indications for cesarean section in a teaching hospital in China. Eur J Obstetr Gynecol Reprod Biol.

[34] Wei J, Wang H, Yang X, Dong M, Wang Z (2010). Altered gene profile of placenta from women with intrahepatic cholestasis of pregnancy. Arch Gynecol Obstet.

[35] Dong W, Xu D, Hu Z, He X, Guo Z, Jiao Z, Yu Y, Wang H (2018). Low-functional programming of the CREB/BDNF/TrkB pathway mediates cognitive impairment in male offspring after prenatal dexamethasone exposure. Toxicol Lett.

[36] Committee Opinion No. 713: Antenatal corticosteroid therapy for fetal maturation. Obstet Gynecol 2017;130(2):e102-e1e9. 10.1097/AOG.0000000000002237 28742678

[37] Kemp MW, Newnham JP, Challis JG, Jobe AH, Stock SJ (2016). The clinical use of corticosteroids in pregnancy. Hum Reprod Update.

[38] Reagan-Shaw S, Nihal M, Ahmad N (2008). Dose translation from animal to human studies revisited. FASEB J.

[39] Zeng H, Jiang Y, Chen P, Fan X, Li D, Liu A, Ma X, Xie W, Liu P, Gonzalez FJ, Huang M, Bi H (2017). Schisandrol B protects against cholestatic liver injury through pregnane X receptors. Br J Pharmacol.

[40] Li H, van Ravenzwaay B, Rietjens IM, Louisse J (2013). Assessment of an in vitro transport model using BeWo b30 cells to predict placental transfer of compounds. Arch Toxicol.

[41] Liu M, Chen B, Pei L, Zhang Q, Zou Y, Xiao H, Zhou J, Chen L, Wang H (2018). Decreased H3K9ac level of StAR mediated testicular dysplasia induced by prenatal dexamethasone exposure in male offspring rats. Toxicology..

[42] Egerman RS, Pierce WF, Andersen RN, Umstot ES, Carr TL, Sibai BM (1997). A comparison of the bioavailability of oral and intramuscular dexamethasone in women in late pregnancy. Obstet Gynecol.

[43] Martineau M, Papacleovoulou G, Abu-Hayyeh S, Dixon PH, Ji H, Powrie R, Larson L, Chien EK, Williamson C (2014). Cholestatic pregnancy is associated with reduced placental 11betaHSD2 expression. Placenta..

[44] Tan Y, Liu J, Deng Y, Cao H, Xu D, Cu F, Lei Y, Magdalou J, Wu M, Chen L, Wang H (2012). Caffeine-induced fetal rat over-exposure to maternal glucocorticoid and histone methylation of liver IGF-1 might cause skeletal growth retardation. Toxicol Lett.

[45] Wen Y, Li J, Tan Y, Qin J, Xie X, Wang L, Mei Q, Wang H, Magdalou J, Chen L (2014). Angelica Sinensis polysaccharides stimulated UDP-sugar synthase genes through promoting gene expression of IGF-1 and IGF1R in chondrocytes: promoting anti-osteoarthritic activity. PLoS One.

[46] Pavek P, Smutny T (2014). Nuclear receptors in regulation of biotransformation enzymes and drug transporters in the placental barrier. Drug Metab Rev.

[47] Halilbasic E, Claudel T, Trauner M (2013). Bile acid transporters and regulatory nuclear receptors in the liver and beyond. J Hepatol.

[48] Garcia-Canaveras JC, Donato MT, Castell JV, Lahoz A (2012). Targeted profiling of circulating and hepatic bile acids in human, mouse, and rat using a UPLC-MRM-MS-validated method. J Lipid Res.

[49] WHO Recommendations on Interventions to Improve Preterm Birth Outcomes. Geneva: World Health Organization; 2015. 26447264

[50] WHO ACTION Trials Collaborators, Oladapo OT, Vogel JP, Piaggio G, Nguyen MH, Althabe F, Gülmezoglu AM, Bahl R, et al. Antenatal dexamethasone for early preterm birth in low-resource countries. N Engl J Med. 2020;383(26):2514–25. Advance online publication. 10.1056/NEJMoa2022398 PMC7660991 33095526

[51] Rohwer AC, Oladapo OT, Hofmeyr GJ (2020). Strategies for optimising antenatal corticosteroid administration for women with anticipated preterm birth. Cochrane Database Syst Rev.

[52] Razaz N, Skoll A, Fahey J, Allen VM, Joseph KS (2015). Trends in optimal, suboptimal, and questionably appropriate receipt of antenatal corticosteroid prophylaxis. Obstet Gynecol.

[53] Jordan BK, Schilling D, McEvoy CT (2018). The window of improved neonatal respiratory compliance after rescue antenatal steroids. J Perinatol: official journal of the California Perinatal Association.

[54] Roberts D, Brown J, Medley N, Dalziel SR (2017). Antenatal corticosteroids for accelerating fetal lung maturation for women at risk of preterm birth. Cochrane Database Syst Rev..

[55] Sokol RJ, Straka MS, Dahl R, Devereaux MW, Yerushalmi B, Gumpricht E, Elkins N, Everson G (2001). Role of oxidant stress in the permeability transition induced in rat hepatic mitochondria by hydrophobic bile acids. Pediatr Res.

[56] Liu Y, Havinga R, VAN DER Leij FR, Boverhof R, Sauer PJ, Kuipers F (2008). Dexamethasone exposure of neonatal rats modulates biliary lipid secretion and hepatic expression of genes controlling bile acid metabolism in adulthood without interfering with primary bile acid kinetics. Pediatr Res.

[57] Li XQ, Zhu P, Myatt L, Sun K (2014). Roles of glucocorticoids in human parturition: a controversial fact?. Placenta..

[58] Leazer TM, Klaassen CD (2003). The presence of xenobiotic transporters in rat placenta. Drug Metab Dispos.

[59] Mennone A, Soroka CJ, Cai SY, Harry K, Adachi M, Hagey L (2006). Mrp4−/− mice have an impaired cytoprotective response in obstructive cholestasis. Hepatology (Baltimore).

[60] Hirvioja ML, Tuimala R, Vuori J (1992). The treatment of intrahepatic cholestasis of pregnancy by dexamethasone. Br J Obstet Gynaecol.

[61] Li T, Chiang JY (2013). Nuclear receptors in bile acid metabolism. Drug Metab Rev.

[62] Pathak P, Liu H, Boehme S, Xie C, Krausz KW, Gonzalez F, Chiang JYL (2017). Farnesoid X receptor induces Takeda G-protein receptor 5 cross-talk to regulate bile acid synthesis and hepatic metabolism. J Biol Chem.

[63] Hartmann P, Hochrath K, Horvath A, Chen P, Seebauer CT, Llorente C (2018). Modulation of the intestinal bile acid/farnesoid X receptor/fibroblast growth factor 15 axis improves alcoholic liver disease in mice. Hepatology (Baltimore).

[64] Narang VS, Fraga C, Kumar N, Shen J, Throm S, Stewart CF, Waters CM (2008). Dexamethasone increases expression and activity of multidrug resistance transporters at the rat blood-brain barrier. Am J Physiol Cell Physiol.

[65] Petropoulos S, Gibb W, Matthews SG (2011). Glucocorticoid regulation of placental breast cancer resistance protein (Bcrp1) in the mouse. Reprod Sci (Thousand Oaks).

[66] Mauvais-Jarvis F, Arnold AP, Reue K (2017). A guide for the design of pre-clinical studies on sex differences in metabolism. Cell Metab.

[67] Iyer A, Kauter K, Brown L (2011). Gender differences in metabolic syndrome: a key research issue?. Endocrine Metab Immune Disord Drug Targets.

[68] Chen Z, Zhao Z, Li Y, Zhang X, Li B, Chen L, Wang H (2018). Course-, dose-, and stage-dependent toxic effects of prenatal dexamethasone exposure on fetal articular cartilage development. Toxicol Lett.

[69] Zhang X, Shang-Guan Y, Ma J, Hu H, Wang L, Magdalou J, Chen L, Wang H (2016). Mitogen-inducible gene-6 partly mediates the inhibitory effects of prenatal dexamethasone exposure on endochondral ossification in long bones of fetal rats. Br J Pharmacol.

[70] Lv F, Wan Y, Chen Y, Pei L, Luo D, Fan G, Luo M, Xu D, Wang H (2018). Prenatal dexamethasone exposure induced ovarian developmental toxicity and transgenerational effect in rat offspring. Endocrinology..

